# Elevated circulating tumor cells and squamous cell carcinoma antigen levels predict poor survival for patients with locally advanced cervical cancer treated with radiotherapy

**DOI:** 10.1371/journal.pone.0204334

**Published:** 2018-10-10

**Authors:** Yue-Feng Wen, Tian-Tian Cheng, Xiao-Long Chen, Wen-Jin Huang, Hai-Hua Peng, Tong-Chong Zhou, Xiao-Dan Lin, Li-Si Zeng

**Affiliations:** 1 Department of Radiotherapy, Affiliated Cancer Hospital & Institute of Guangzhou Medical University, Guangdong, China; 2 Department of Good Clinical Practice Center, Affiliated Cancer Hospital & Institute of Guangzhou Medical University, Guangdong, China; 3 Department of Orthopedics, Guangdong Work Injury Rehabilitation Hospital, Guangdong, China; 4 Department of Abdominal Surgery, Affiliated Cancer Hospital & Institute of Guangzhou Medical University, Guangdong, China; National Cancer Center, JAPAN

## Abstract

**Objective:**

To evaluate the prognostic effects of combining serum circulating tumor cells (CTCs) and squamous cell carcinoma antigen (SCC-Ag) levels on patients with locally advanced cervical cancer treated with radiotherapy.

**Methods:**

Ninety-nine patients with locally advanced cervical cancer ([FIGO] stage IIB-IVA) undergoing radiotherapy (RT) or concurrent chemoradiotherapy (CCRT) were identified. The association between serum CTC level and clinicopathological parameters was examined. Univariate and multivariate survival analyses were performed by using Cox’s proportional hazards regression model.

**Results:**

Elevated CTC and SCC-Ag levels were significantly associated with poor disease-free survival (DFS). Multivariate analysis suggest that serum CTC level, FIGO stage and serum SCC-Ag level were independent prognostic factors for two-year DFS. When CTC and SCC-Ag levels were combined into a new risk model to predict disease progression of cervical cancer patients, it performed a significantly better predictive efficiency compared with either biomarker alone.

**Conclusion:**

Serum CTC and SCC-Ag levels are potentially useful biomarkers for prediction of prognosis in locally advanced cervical cancer patients and their combination significantly improves predictive ability for survival in locally advanced cervical cancer patients.

## Introduction

According to a recently published study by the GLOBOCAN on the research of cancer, cervical cancer is the fourth most common cancer in women, with approximately 266,000 cancer-related deaths worldwide in 2012[1]. Concurrent chemoradiotherapy (CCRT) has become the primary treatment modality for the patients with International Federation of Gynecology and Obstetrics [FIGO] stage IIB-IVA cervical cancer based on the encouraging results from several randomized trials published in 1999[2–5]. Unfortunately, about 30–50% patients after definitive radiotherapy (RT) will develop local recurrence or distant metastatic disease[6–8]. Therefore, it is significantly important to identify prognostic factors in patients with locally advanced cervical cancer treated with RT or CCRT.

Squamous cell carcinoma antigen (SCC-Ag) is the most widely used and reliable tumor marker for the diagnosis[9], monitoring[10]and prediction of cervical cancer prognosis[7]. Recently, several high-risk factors in clinical practice, including advanced stage, bulky tumor size, positive pelvic lymph node metastasis, and high serum SCC-Ag levels were found to be associated with poor prognosis in cervical cancer patients[11]. However, a subgroup of high-risk patients does not benefit from adjuvant chemotherapy, suggesting that current selection criteria for high-risk patients seems insufficient. Therefore, we sought to identify additional markers that could complement SCC.

Circulating tumor cells (CTCs) are rare tumor cells that are shed from primary or metastatic tumors detected in peripheral blood[12]. CTC has been identified as a significant prognostic biomarker for breast cancer[13], colorectal cancer[13], prostate cancer[14], lung cancer[15], and ovarian cancer[16]. A pooled analysis demonstrated that around 50% metastatic breast cancer patients had baseline serum CTCs levels ≥5 CTCs/7.5 ml, and these patients had poorer progression-free survival (PFS) and overall survival (OS) compared with patients with a low CTC count(<5 CTCs/7.5 ml)[17]. However, the impact of CTC on prognosis for cervical cancer patients has been unclear so far. Therefore, we conducted this retrospective study to identify CTC as a prognostic factor for patients with locally advanced cervical cancer treated with definitive RT. We also evaluated whether CTC combined with SCC levels may refine the prognostic stratification for locally advanced cervical cancer patients.

## Materials and methods

### Patients’ characteristics

We retrospectively reviewed 99 consecutive patients with locally advanced cervical cancer ([FIGO] stage IIB-IVA) undergoing radiotherapy (RT) or concurrent chemoradiotherapy (CCRT) from November 2012 to December 2014 at the Affiliated Cancer Hospital & Institute of Guangzhou Medical University, China. This study was reviewed and approved by the institutional review board and ethics committee of Affiliated Cancer Hospital & Institute of Guangzhou Medical University. All participants gave their written informed consent.

### Clinical management

The pre-treatment evaluations included a complete patient history, physical examinations, haematology and biochemistry profiles, SCC-Ag, CTC, colposcopy, chest X-ray, abdominal sonography, electrocardiogram, magnetic resonance imaging (MRI), computed tomographies (CTs), and whole-body bone scan. All the patients were treated with external beam radiotherapy (EBRT) and brachytherapy. The EBRT dose was 45~50 Gy delivered in 25 fractions (5 days per week, one fraction per day and 1.8~2.0 Gy per fraction) by using a conventional four-field box technique. The radiation field included the primary tumor, uterus, pelvic lymph node and the paracervical, parametrial, and uterosacral regions. High-dose rate intracavitary brachytherapy was delivered two fractions per week with a fraction dose of 5.0 Gy at point A for 4~6 fractions. A month after the completion of RT or CCRT, all patients were given pathological examination and pelvic MRI.

### Concurrent chemoradiotherapy

The regimens for CCRT consisted of intravenous infusion of weekly cisplatin or cisplatin combined with docetaxel administered every 3 weeks during radiotherapy.

### Follow-up

The first patient follow-up was administered at one month after RT or CCRT completion, then every 3~6 months for the first two years, and every 6–12 months thereafter. The follow-up intervals can be assessed more frequently for patients suspected of having recurrent or metastatic diseases.

### Enrishment and identification of CTCs

Peripheral blood samples were collected via venipuncture before radiotherapy and processed within 24 hours after collection. To avoid bias, all blood samples collection, enrichment, and result reading were blindly performed by different personal. The strategy of enrichment of cervical cancer CTCs was essentially similar to the one that was previously published [18]. In brief, 3.2 ml of collected peripheral blood was firstly processed by lysis of RBC. Then, the residue cell pellet was resuspended in PBS and subsequently incubated with anti-CD45 monoclonal antibody-coated magnetic beads for 30 min, followed by the separation of magnetic beads using a magnetic stand (Promega, Madison, WI, USA). Supernatants were subsequently subjected to immunofluorescence analysis. The identification of enriched cervical cancer CTCs was performed by negative enrichment and immune fluorescence in situ hybridization (NEimFISH) which combined the FISH with chromosome 8 (orange) centromere probe (Abbott Molecular Diagnostics, Des Plaines, IL, USA) and anti-CD45 monoclonal antibody (red). In brief, the probe CEP8 and specimen were hybridized at 37°C for 20 min in hybridizer (DAKO). Subsequently, they were washed in 50% formamide at 43°C for 15 min, then immersed into 2*SSC and gradient alcohol again. At last, the specimens were washed twice with 0.2% BSA and incubated with the CD45 mixture/2%BSA conjugated to Alexa Fluor 594 (Invitrogen) for 1 h. Afterward, they were washed again with 0.2% BSA. Finally, the specimens were covered with DAPI which contained Vectashield mounting medium. The area of the fixed sample should be observed entirely along ‘‘S” track with a microscope (Nikon). Positive CTCs must meet hyperdiploid CEP8+/DAPI+/CD45-.

### Measurement of concentration of serum SCC-Ag

Serum SCC-Ag levels were measured by using microparticle enrymeimmunoassay (MEIA) with a commercially available kit (Imx, Abbott Diagnostics, Abbott Park, IL, USA). The normal upper limit for SCC-Ag was 1.5 ng/mL.

### Statistical analysis

The association between pretherapeutic serum CTC level and clinicopathological parameters were performed by the chi-square test. Receiver operative characteristic (ROC) curve was used to determine the optimal cutoff value of tumor markers for survival. According to the cutoff value, the cervical cancer patients were divided into two groups, CTC negative group and CTC positive group. Disease-free survival (DFS) was defined as the time from the date of definite diagnosis to the date of disease progression (local recurrence or distant metastasis) or censored at the date of last follow-up. Survival times of patients still alive or dead as a result of other causes than cancer were censored with the last follow-up date. DFS was calculated by the Kaplan–Meier method. The differences of survival were compared with the log-rank test. Univariate and multivariate analysis were performed using Cox’s proportional hazards regression model with a forward stepwise procedure (the entry and removal probabilities were 0.05 and 0.10, respectively). Analyses were performed using the statistical software package SPSS 20.0 (SPSS, Chicago, IL) and Graph Pad Prism for windows, version 6 (Graph Pad Prism, San Diego, CA, USA). A two-sided P-value less than 0.05 was considered statistically significant.

## Results

### Patients’ characteristics

**Fig 1:**
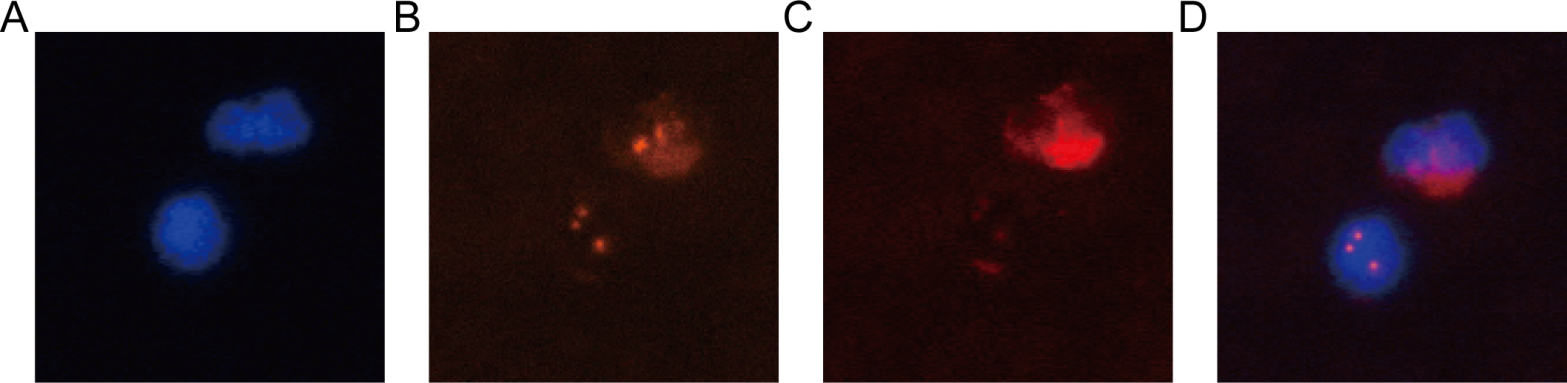
Identification of CTCs by imFISH. **(A)** DAPI,blue; **(B)** CEP8,orange; **(C)** CD45, red; **(D)** merge. It shows CTCs as triploid CEP8+/ DAPI+/CD45- and WBC as diploid CEP8+/DAPI+/CD45+ **(D)**.

**Fig 2:**
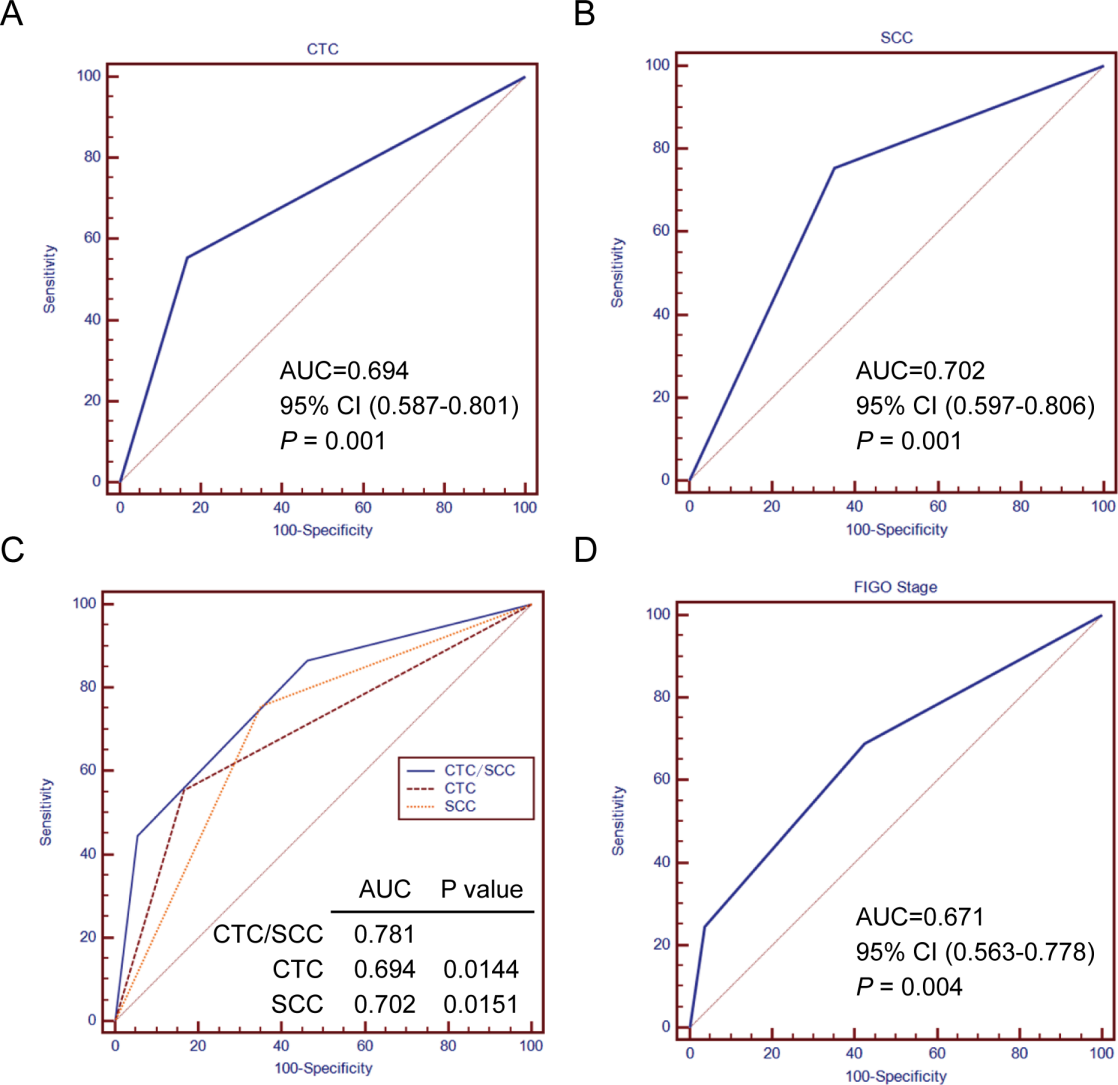
Comparison of the survival prediction of patients with cervical cancer by serum CTC or SCC expression levels alone and the combination of both. **(A)** and **(B)** ROC curve analysis of the optimal cutoff value of serum CTC or SCC level for distinguishing disease-free survival (DFS) from patients with cervical cancer. AUC was 0.694 for serum CTC (P = 0.001) and 0.702 for serum SCC (P = 0.001). **(C)** ROC analysis compares the survival prediction of patients with cervical cancer by serum CTC or SCC expression levels alone and the combination of both. The result shows that the area under the curve (AUC) of the combined serum CTC and SCC expression is the largest among the three predictors, which demonstrates that predictive accuracy of the combined risk model is better than those of FIGO stage and serum CTC or SCC alone. **(D)** ROC curve analysis of the predictive accuracy for DFS of patients with cervical cancer by FIGO staging system (AUC = 0.671, P = 0.004).

The study included a total of 99 cervical cancer patients with FIGO stage IIB-IVA. The clinicopathological characteristics of the study cohort were shown in Table 1. The range of CTCs detected in this study was 0–30 CTCs/3.2 ml. More than 2 CTCs/3.2 ml was considered as positive. The positive rate of CTCs was 45.5% (45/99) in our study. Positive cervical cancer cell and white blood cell (WBC) were depicted in Fig 1. The optimal cutoff values of serum CTC and SCC-Ag level with the best discriminatory power were 2.5 CTCs/3.2 ml blood and 4.5 ng/ml respectively according to ROC curve analysis (Fig 2). For optimization for their potential acceptance and application in clinical practice, we chose the nearest integer of 3 CTCs/3.2 ml blood and 5 ng/ml as the final cutoff point. There were 65 and 34 patients assigned to the CTC negative group (CTC < 3 CTCs/3.2 ml) and CTC positive group (CTC ≥ 3 CTCs/3.2 ml) respectively (65.7% vs. 34.3%).

**Table 1:** The clinicopathological parameters of patients with cervical cancer.

Clinicopathological parameters	N (%)
Age (years)	
< 45	20 (20.2%)
≥ 45	79 (79.8%)
Tumor size (cm)	
< 4	55 (55.6%)
≥ 4	44 (44.4%)
Histological types	
Squamous cell carcinoma	86 (86.9%)
Adenocarcinoma	12 (12.1%)
Adenosquamous carcinoma	1 (1.0%)
FIGO stage	
II	45 (45.5%)
III	41 (41.4%)
IV	13 (13.1%)
Squamous cell carcinoma antigen (ng/ml)	
< 5	46 (46.5%)
≥ 5	53 (53.5%)
Parametrial extension	
No	40 (40.4%)
Yes	59 (59.6%)
CTC (/3.2ml)	
< 3	65 (65.7%)
≥ 3	34 (34.3%)

NOTE: CTC, circulating tumor cells, FIGO, International Federation of Gynecology and Obstetrics.

### Association between serum CTC level and clinicopathological characteristics

The pretreatment serum CTC level was associated with SCC-Ag. No association between CTC and other clinicopathological characteristics was found (Table 2).

**Table 2:** Correlation between CTC and clinicopathological parameters in cervical cancer patients.

Parameters	N	CTC		*P* value
		Negative (N, %)	Positive (N, %)
Age (years)				
< 45	20	15 (75.0%)	5 (25.0%)	0.325
≥ 45	79	50 (63.3%)	29 (36.7%)	
Tumor size (cm)				
< 4	55	33 (60.0%)	22 (40.0%)	0.185
≥ 4	44	32 (72.7%)	12 (27.3%)	
Histological types				
Squamous cell carcinoma	86	57 (66.3%)	29 (33.7%)	0.381
Adenocarcinoma	12	8 (66.7%)	4 (33.3%)	
Adenosquamous carcinoma	1	0 (0.0%)	1 (100.0%)	
FIGO stage				
II	45	35 (77.8%)	10 (22.2%)	0.067
III	41	23 (56.1%)	18 (43.9%)	
IV	13	7 (53.8%)	6 (46.2%)	
Squamous cell carcinoma antigen (ng/ml)				
< 5	46	35 (76.1%)	11 (23.9%)	**0.042**
≥ 5	53	30 (56.6%)	23 (43.4%)	
Parametrial extension				
No	40	25 (62.5%)	15 (37.5%)	0.586
Yes	59	40 (67.8%)	19 (32.2%)	

NOTE: CTC, circulating tumor cells, FIGO, International Federation of Gynecology and Obstetrics. The bold type represents *P* values smaller than 0.05.

### Association between serum CTC level and prognosis

**Fig 3:**
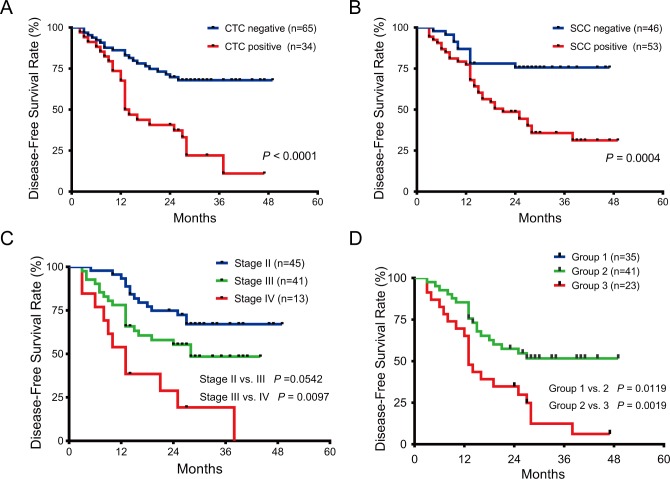
Survival prediction of patients with cervical cancer by FIGO staging system and the expression level of serum CTC or SCC. **(A)** The disease-free survival (DFS) curves of patients with positive and negative expression level of serum CTC (P < 0.0001). **(B)** The DFS curves of patients with positive and negative expression level of serum SCC (P = 0.0004). **(C)** DFS outcomes of patients with cervical cancer were predicted by FIGO staging system (Stage I vs. II, P = 0.0542; Stage II vs. III, P = 0.0097). **(D)** DFS outcomes of patients with cervical cancer were predicted by the combined serum CTC and SCC expression levels, which show that the combined risk model significantly improves survival prediction of patients (Group I, II and III represent low-risk, intermediate-risk and high-risk group, respectively. Group I vs. II, P = 0.0119; Group II vs. III, P = 0.0019).

In univariate survival analysis, elevated CTC level, was strongly associated with poorer DFS of cervical cancer patients with HR of 3.354 (95% confidence interval [CI], 1.850–6.080) (Table 3). The two-year DFS rates for patients with serum CTC levels < 3 CTCs/3.2 ml and ≥ 3 CTCs/3.2 ml were 71.3% and 40.6%, respectively (P < 0.0001) (Fig 3). In addition to serum CTC level, FIGO stage and serum SCC-Ag level were significantly associated with DFS of cervical cancer patients (Fig 3). Multivariate analysis suggested that elevated CTC level remained as a highly significant predictor of DFS. Patients in the CTC positive group had 2.425 (95% CI, 1.313–4.477, P = 0.005) times the risk of progression compared with those in the CTC negative group. Moreover, advanced FIGO stage and elevated SCC-Ag level were also associated with poorer DFS in multivariable survival analyses (Table 3).

**Table 3:** Univariate and multivariate analysis of the effects of CTC and clinicopathological characteristics on disease-free survival in patients with cervical cancer.

Clinicopathological characteristics	Univariate analysis		Multivariate analysis	
	HR (95% CI)	*P* value	HR (95% CI)	*P* value
Age (≥ 45 years vs. < 45 years)	0.941 (0.466–1.901)	0.865		
Tumor size (≥ 4 cm vs. < 4 cm)	1.108 (0.617–1.991)	0.731		
Histological types (Adenosqua vs. Adeno vs. Squa)	1.527 (0.740–3.152)	0.252		
FIGO stage (IV vs. III vs. II)	2.202 (1.458–3.326)	**0.000**	1.671 (1.109–2.517)	**0.014**
SCC-Ag (≥5 ng/ml vs. < 5 ng/ml)	3.125 (1.581–6.175)	**0.001**	2.338 (1.162–4.705)	**0.017**
Parametrial extension (Yes vs. No)	1.042 (0.574–1.893)	0.892		
CTC status (Positive vs. Negative)	3.354 (1.850–6.080)	**0.000**	2.425 (1.313–4.477)	**0.005**

NOTE: CTC, circulating tumor cells; Adenosqua, Adenosquamous carcinoma; Adeno, Adenocarcinoma; Squa, Squamous cell carcinoma; FIGO, International Federation of Gynecology and Obstetrics; SCC-Ag, Squamous cell carcinoma antigen; HR, hazard ratio; CI, confidence interval. The bold type represents *P* values smaller than 0.05.

### Construction of a new molecular risk model combining CTC with SCC-Ag levels

For elevated CTC and SCC-Ag levels were strong predictors of DFS, we hypothesize that combination of these 2 biomarkers would improve their predictive efficiency for survival. Then, the new combined risk model performed significantly better than CTC or SCC-Ag level alone (Fig 2). Therefore, we divided the entire study cohort into 3 risk groups by calculating the new combined risk score as the sum of CTC score (0 or 1) and SCC-Ag score (0 or 1) for each case. Patients with low- (score 0), intermediate- (score 1) or high-risk (score 2) had significantly different 2-year DFS rates, 82.4%, 57.4% and 34.8%, respectively (Fig 3). Multivariate analysis suggest that the new combined risk model was an independent prognostic factor for DFS in cervical cancer patients (Table 4). Furthermore, the new combined risk model had slightly better predictive efficiency for DFS of cervical cancer patients compared with FIGO stage though there was no significantly difference between the Area Under Curve (AUC) value (AUC, 0.781 vs. 0.671, Fig 2). Therefore, the new combined risk model may be a useful biomarker for the more individualized treatment of the cervical cancer patients.

**Table 4:** Univariate and multivariate analysis of the effects of CTC/SCC and disease-free survival in patients with cervical cancer.

Clinicopathological characteristics	Univariate analysis		Multivariate analysis	
	HR (95% CI)	*P* value	HR (95% CI)	*P* value
FIGO stage (IV vs. III vs. II)	2.202 (1.458–3.326)	**0.000**	1.671 (1.110–2.516)	**0.014**
CTC/SCC combination status (Low vs. Intermediate vs. High)	2.711 (1.790–4.106)	**0.000**	2.386 (1.548–3.678)	**0.000**

NOTE: CTC, circulating tumor cells; SCC-Ag, Squamous cell carcinoma antigen; FIGO, International Federation of Gynecology and Obstetrics; HR, hazard ratio; CI, confidence interval. The bold type represents *P* values smaller than 0.05.

## Discussion

Although FIGO stage is the most important clinical prognostic indicators for cervical cancer patients, many patients with the same FIGO stage have different treatment outcomes due to tumor heterogeneity, suggesting that the FIGO stage system urgently needs to be improved by additional prognostic factors such as tumor biomarkers for recurrence and metastasis. To the best of our knowledge, this is the first study investigating the role of CTC alone or in combination with SCC as independent prognostic factors for disease progression in locally advanced cervical cancer patients treated with radiotherapy. In the present study, our results demonstrated that CTC and SCC-Ag individually or in combination were associated with DFS of locally advanced cervical cancer patients. In addition, the predictive efficiency could be significantly improved when CTC and SCC-Ag were combined into a new risk model to predict disease progression of cervical cancer patients compared with either biomarker alone, indicating that the combination of these 2 biomarkers might help to improve the prognostic stratification and guide the individual treatment for cervical cancer patients in the future.

CTCs are identified as tumor cells shed from the primary solid tumor into blood circulation. CellSearch is the only CTC detection technique that has been approved for clinical use by the Food and Drug Administration. However, CellSearch has a limit sensitivity and specificity due to its dependence on epithelial cell adhesion molecule (EpCAM) enrichment technique. Brigitte et al. reported that the positive rate of CTCs detected by the Cellsearch system was only 21.5% in 2026 patients with early breast cancer before adjuvant chemotherapy [19]. In this study, we found that the CTCs positive rate was 45.5% in patients with locally advanced cervical cancer which was within the CTCs detection rates ranging from 35% to 65% reported by other investigators [20].

Bayarri et al. found that the detection rate of CTCs in 56 patients with stage I-IIIA non-small cell lung cancer (NSCLC) was 51.8% by immunocytochemical and related with the disease free survival for NSCLC patients [19, 21–23]. According to ROC curve analysis, the optimal cutoff value of 2.5 CTCs/3.2 ml blood for imFISH yielded sensitivity of 55.6% and specificity of 83.3% in our study. Chen YY, et al found that the sensitivity and specificity of imFISH combining CEP7 with CEP8 in diagnosis of primary lung cancer was 84% and 97.6%, respectively [18].Thus, it seems helpful to improve the sensitivity and specificity of the imFISH technology by using combined multiple chromosome probes.

Moreover, we also observed that CTC, SCC-Ag and FIGO stage were significant independent prognostic factors for DFS of locally advanced cervical cancer patients. Elevated serum CTC and SCC-Ag level predicted poorer survival for cervical cancer patients. Our results were in line with the results from several previous studies [13, 20, 24, 25]. Lucci et al. reported that CTC detected by the CellSearch system was an independent prognostic marker for OS and PFS in patients with non-metastatic breast cancer[20]. Bork et al. revealed that CTC count ≥1 CTC/7.5 ml blood was significantly associated with worse OS in the non-metastatic colorectal cancer[13]. Dorsey et al. found that CTC counts were correlated with radiotherapy response in patients with localized non-small-cell lung cancer (NSCLC) undergoing definitive radiotherapy [24]. A meta-analysis confirmed that the presence of CTCs indicated a poor prognosis in NSCLC patients[25].

Serum SCC-Ag levels were demonstrated to be associated with the FIGO stage[9, 26, 27], lymph node metastasis, recurrence[28], and prediction of cervical cancer patients’ prognosis [29–31]. Scambia et al. revealed that high SCC-ag levels was associated with pelvic lymph node metastasis in patients with early-stage cervical cancer treated with radical surgery [10]. Oh et al. reported that higher pretreatment SCC-Ag levels predicted poorer survival[32].

However, the molecular mechanism by which the combination of both biomarkers improves the prognostic stratification for cervical cancer patients is still unclear. It is probably that CTC and SCC promote the progression of cervical cancer through taking part in the tumor signaling pathways or influencing the tumor biological behaviors. Epithelial–mesenchymal transition (EMT) is a complex process that the epithelial cell loses differentiation and increases motility via rearrangements of cellular contact junctions and eventually the loss of cell adhesion. EMT might involve the formation of CTC by increasing the ability of the tumor cell to migrate and effecting the cancer aggressiveness[33]. Therefore, further studies are required to reveal the molecular mechanisms of tumor cell dissemination.

As a retrospective study, our study was limited by biases such as lack of random assignment, and patient’s incomplete data acquisition. Therefore, larger trials with longer follow-up times are promptly needed to further testify whether detection of serum CTC and SCC-Ag levels could improve the prognostic stratification and guide the individual treatment for cervical cancer patients.

## Conclusion

In conclusion, our study reveals that elevated CTC and SCC-Ag levels individually or in combination are significantly associated with poorer DFS of locally advanced cervical cancer patients, indicating that they are potentially useful biomarkers for prediction of prognosis in cervical cancer patients. Further studies are required to reveal the molecular mechanism by which the combination of both biomarkers improves the prognostic stratification for cervical cancer patients.

## Ethical approval

All procedures performed in studies involving human participants were in accordance with the ethical standards of the institutional and/or national research committee and with the 1964 Helsinki declaration and its later amendments or comparable ethical standards. There was no disagreements among all participants included in the study.

## Supporting information

S1 Table Clinicopathological parameters and prognosis of 99 cervical cancer patients. (DOCX)
