# Supplementary material for: Elevated circulating tumor cells and squamous cell carcinoma antigen levels predict poor survival for patients with locally advanced cervical cancer treated with radiotherapy
Source: PLoS One. 2018 Oct 10;13(10):e0204334. doi: 10.1371/journal.pone.0204334 (PMC6179236; doi:10.1371/journal.pone.0204334)
Supplement: S1 Table — (DOCX) [file pone.0204334.s001.docx]

Supporting information

Table S1. Clinicopathological parameters and prognosis of 99 cervical cancer patients

| case | age | Tsize | patho | stage | SCC | PE | CTC | CSC | progress | DFS |
| --- | --- | --- | --- | --- | --- | --- | --- | --- | --- | --- |
| 67 | 0 | 1 | 1 | 4 | 1 | 0 | 0 | 1 | 1 | 25 |
| 38 | 0 | 0 | 1 | 4 | 0 | 1 | 0 | 0 | 0 | 14 |
| 9 | 0 | 0 | 1 | 3 | 1 | 0 | 0 | 1 | 0 | 28 |
| 98 | 0 | 0 | 1 | 2 | 0 | 1 | 0 | 0 | 0 | 33 |
| 31 | 0 | 1 | 2 | 2 | 1 | 1 | 1 | 2 | 1 | 27 |
| 69 | 0 | 0 | 1 | 2 | 0 | 1 | 0 | 0 | 0 | 26 |
| 70 | 0 | 1 | 1 | 2 | 1 | 1 | 0 | 1 | 0 | 40 |
| 37 | 0 | 0 | 1 | 3 | 0 | 0 | 0 | 0 | 1 | 13 |
| 79 | 0 | 0 | 2 | 2 | 0 | 1 | 0 | 0 | 1 | 10 |
| 20 | 0 | 1 | 1 | 2 | 0 | 1 | 0 | 0 | 0 | 29 |
| 89 | 0 | 1 | 1 | 3 | 1 | 0 | 0 | 1 | 0 | 44 |
| 84 | 0 | 1 | 1 | 4 | 1 | 0 | 1 | 2 | 1 | 38 |
| 52 | 0 | 0 | 1 | 2 | 0 | 1 | 0 | 0 | 0 | 46 |
| 74 | 0 | 1 | 2 | 2 | 0 | 1 | 0 | 0 | 0 | 37 |
| 54 | 0 | 1 | 1 | 4 | 1 | 0 | 0 | 1 | 1 | 21 |
| 2 | 0 | 1 | 1 | 3 | 1 | 1 | 0 | 1 | 1 | 15 |
| 101 | 0 | 0 | 1 | 3 | 1 | 0 | 1 | 2 | 1 | 13 |
| 53 | 0 | 0 | 1 | 2 | 1 | 1 | 0 | 1 | 0 | 29 |
| 28 | 0 | 0 | 1 | 3 | 1 | 1 | 1 | 2 | 1 | 3 |
| 92 | 0 | 0 | 1 | 3 | 1 | 0 | 1 | 2 | 1 | 4 |
| 14 | 1 | 0 | 1 | 2 | 1 | 1 | 0 | 1 | 0 | 29 |
| 55 | 1 | 0 | 1 | 2 | 1 | 1 | 0 | 1 | 1 | 14 |
| 41 | 1 | 1 | 1 | 2 | 1 | 1 | 0 | 1 | 1 | 18 |
| 95 | 1 | 1 | 1 | 4 | 1 | 1 | 1 | 2 | 1 | 3 |
| 72 | 1 | 0 | 2 | 2 | 0 | 1 | 0 | 0 | 0 | 30 |
| 13 | 1 | 1 | 2 | 2 | 0 | 1 | 0 | 0 | 0 | 27 |
| 18 | 1 | 0 | 1 | 3 | 0 | 1 | 0 | 0 | 1 | 7 |
| 94 | 1 | 0 | 1 | 3 | 0 | 0 | 1 | 1 | 0 | 28 |
| 24 | 1 | 0 | 1 | 2 | 1 | 1 | 0 | 1 | 0 | 49 |
| 19 | 1 | 0 | 1 | 2 | 1 | 1 | 1 | 2 | 1 | 13 |
| 86 | 1 | 0 | 1 | 2 | 0 | 1 | 0 | 0 | 0 | 33 |
| 50 | 1 | 1 | 1 | 2 | 0 | 1 | 0 | 0 | 0 | 26 |
| 3 | 1 | 0 | 2 | 3 | 0 | 1 | 1 | 1 | 1 | 13 |
| 58 | 1 | 1 | 1 | 3 | 0 | 0 | 0 | 0 | 0 | 44 |
| 11 | 1 | 0 | 1 | 2 | 1 | 1 | 0 | 1 | 0 | 28 |
| 68 | 1 | 1 | 1 | 2 | 1 | 1 | 0 | 1 | 0 | 28 |
| 80 | 1 | 1 | 1 | 3 | 1 | 1 | 1 | 2 | 1 | 28 |
| 77 | 1 | 0 | 1 | 2 | 0 | 1 | 0 | 0 | 0 | 37 |
| 96 | 1 | 0 | 1 | 2 | 0 | 1 | 0 | 0 | 0 | 39 |
| 90 | 1 | 1 | 1 | 3 | 0 | 0 | 0 | 0 | 0 | 37 |
| 39 | 1 | 0 | 1 | 2 | 0 | 1 | 1 | 1 | 0 | 26 |
| 27 | 1 | 1 | 1 | 3 | 1 | 1 | 0 | 1 | 1 | 8 |
| 30 | 1 | 1 | 1 | 3 | 1 | 0 | 0 | 1 | 0 | 40 |
| 1 | 1 | 1 | 1 | 2 | 1 | 1 | 0 | 1 | 1 | 15 |
| 32 | 1 | 1 | 1 | 3 | 0 | 0 | 1 | 1 | 0 | 27 |
| 15 | 1 | 1 | 1 | 3 | 1 | 0 | 0 | 1 | 0 | 14 |
| 44 | 1 | 0 | 1 | 3 | 0 | 0 | 0 | 0 | 0 | 26 |
| 83 | 1 | 1 | 1 | 3 | 0 | 0 | 0 | 0 | 0 | 29 |
| 17 | 1 | 0 | 1 | 3 | 0 | 0 | 1 | 1 | 0 | 13 |
| 59 | 1 | 1 | 1 | 3 | 1 | 0 | 0 | 1 | 0 | 41 |
| 33 | 1 | 0 | 1 | 3 | 0 | 1 | 0 | 0 | 1 | 4 |
| 100 | 1 | 0 | 1 | 2 | 0 | 1 | 0 | 0 | 0 | 33 |
| 5 | 1 | 1 | 1 | 2 | 1 | 1 | 0 | 1 | 0 | 34 |
| 23 | 1 | 0 | 1 | 3 | 1 | 0 | 0 | 1 | 0 | 39 |
| 64 | 1 | 0 | 1 | 2 | 1 | 1 | 1 | 2 | 1 | 16 |
| 40 | 1 | 0 | 1 | 2 | 1 | 1 | 1 | 2 | 1 | 14 |
| 61 | 1 | 1 | 1 | 3 | 1 | 0 | 0 | 1 | 1 | 19 |
| 35 | 1 | 1 | 1 | 2 | 0 | 1 | 0 | 0 | 0 | 31 |
| 73 | 1 | 0 | 2 | 3 | 0 | 0 | 0 | 0 | 0 | 29 |
| 56 | 1 | 1 | 1 | 3 | 0 | 0 | 0 | 0 | 0 | 35 |
| 87 | 1 | 0 | 1 | 3 | 0 | 0 | 1 | 1 | 0 | 33 |
| 97 | 1 | 1 | 1 | 2 | 1 | 1 | 0 | 1 | 1 | 5 |
| 12 | 1 | 0 | 1 | 2 | 1 | 1 | 0 | 1 | 1 | 27 |
| 65 | 1 | 0 | 3 | 2 | 1 | 1 | 1 | 2 | 1 | 12 |
| 82 | 1 | 1 | 1 | 3 | 1 | 1 | 1 | 2 | 1 | 13 |
| 29 | 1 | 1 | 2 | 4 | 1 | 1 | 1 | 2 | 1 | 10 |
| 7 | 1 | 0 | 1 | 4 | 0 | 0 | 0 | 0 | 1 | 9 |
| 81 | 1 | 0 | 1 | 3 | 1 | 0 | 1 | 2 | 1 | 28 |
| 85 | 1 | 0 | 1 | 3 | 0 | 1 | 1 | 1 | 1 | 13 |
| 57 | 1 | 0 | 2 | 2 | 0 | 1 | 1 | 1 | 1 | 13 |
| 48 | 1 | 1 | 1 | 2 | 0 | 1 | 0 | 0 | 0 | 28 |
| 6 | 1 | 0 | 1 | 3 | 0 | 0 | 1 | 1 | 0 | 26 |
| 36 | 1 | 0 | 1 | 3 | 0 | 1 | 1 | 1 | 1 | 10 |
| 78 | 1 | 1 | 1 | 3 | 1 | 0 | 1 | 2 | 1 | 6 |
| 51 | 1 | 0 | 1 | 2 | 0 | 1 | 0 | 0 | 0 | 47 |
| 45 | 1 | 0 | 1 | 2 | 0 | 1 | 0 | 0 | 0 | 28 |
| 10 | 1 | 0 | 1 | 3 | 1 | 0 | 1 | 2 | 0 | 24 |
| 88 | 1 | 0 | 1 | 2 | 0 | 1 | 0 | 0 | 0 | 14 |
| 75 | 1 | 0 | 1 | 2 | 1 | 1 | 1 | 2 | 1 | 25 |
| 21 | 1 | 0 | 1 | 2 | 1 | 1 | 1 | 2 | 1 | 19 |
| 22 | 1 | 1 | 1 | 2 | 0 | 1 | 0 | 0 | 0 | 33 |
| 66 | 1 | 0 | 1 | 2 | 0 | 1 | 0 | 0 | 0 | 37 |
| 8 | 1 | 0 | 1 | 3 | 1 | 0 | 0 | 1 | 1 | 16 |
| 102 | 1 | 0 | 1 | 2 | 1 | 1 | 1 | 2 | 0 | 47 |
| 76 | 1 | 0 | 1 | 3 | 0 | 0 | 0 | 0 | 0 | 44 |
| 47 | 1 | 1 | 1 | 4 | 1 | 0 | 0 | 1 | 1 | 6 |
| 25 | 1 | 0 | 1 | 2 | 1 | 1 | 0 | 1 | 0 | 23 |
| 4 | 1 | 0 | 1 | 3 | 0 | 1 | 1 | 1 | 1 | 9 |
| 63 | 1 | 0 | 1 | 3 | 0 | 0 | 0 | 0 | 1 | 24 |
| 26 | 1 | 1 | 2 | 4 | 1 | 0 | 0 | 1 | 1 | 13 |
| 16 | 1 | 1 | 1 | 4 | 1 | 0 | 1 | 2 | 0 | 27 |
| 62 | 1 | 1 | 1 | 3 | 1 | 0 | 1 | 2 | 1 | 7 |
| 60 | 1 | 1 | 2 | 2 | 0 | 1 | 0 | 0 | 0 | 10 |
| 91 | 1 | 1 | 1 | 4 | 1 | 0 | 0 | 1 | 1 | 3 |
| 34 | 1 | 1 | 1 | 3 | 1 | 0 | 0 | 1 | 0 | 30 |
| 93 | 1 | 1 | 1 | 4 | 1 | 0 | 1 | 2 | 1 | 13 |
| 46 | 1 | 1 | 1 | 4 | 1 | 0 | 1 | 2 | 1 | 8 |
| 43 | 1 | 1 | 1 | 2 | 1 | 1 | 0 | 1 | 0 | 30 |
| 71 | 1 | 0 | 2 | 3 | 0 | 0 | 0 | 0 | 0 | 25 |

age: ≥ 45, 1; < 45, 0

Tsize: Tumor size, ≥ 4, 1; < 4, 0

patho: pathology, squamous cell carcinoma, 1; adenocarcinoma, 2; adenosquamous cell carcinoma, 3 stage: stage II, 2; stage III, 3; stage IV, 4

SCC: ≥ 5, 1; < 5, 0

PE: parametrial extension, yes, 1; no, 0

CTC: ≥ 3, 1; < 3, 0

CSC: CTCSCC, SCC < 5 and CTC < 3, 0; SCC < 5 and CTC ≥3, or SCC ≥ 5 and CTC < 3,1; SCC ≥ 5 and CTC ≥ 3, 2

Progress: yes, 1; no, 0
